# Elizabethkingia Meningoseptica Engodenous Endophthalmitis – a case report

**DOI:** 10.1186/2047-2994-3-35

**Published:** 2014-11-26

**Authors:** Stephanie Ming Young, Gopal Lingam, Paul Anantharajah Tambyah

**Affiliations:** Department of Ophthalmology, National University Health System, 1E Kent Ridge Road, NUHS Tower Block, Singapore, 119228 Singapore; Department of Medicine, National University Health System, 1E Kent Ridge Road, NUHS Tower Block, Singapore, 119228 Singapore

**Keywords:** Elizabethkingia meningoseptica, Endogenous endophthalmitis, Infections of the eye

## Abstract

*Elizabethkingia meningoseptica* is a nosocomial non-fermenting gram-negative bacillus that has an increasing prevalence in health care settings, especially in intensive care environments. While it has long been recognized as a rare but serious cause of neonatal meningitis and sepsis, its role as a cause of ocular pathology is not well-known. We report the first case of *E. meningoseptica* endogenous endophthalmitis caused by bacteraemia by the same organism. In view of its aggressiveness and virulence in the eye, and the high rate of misdiagnosis or missed diagnosis of endogenous endophthalmitis especially given its low incidence, we may wish to consider screening all cases of E. menigoseptica bloodstream infections for endophthalmitis in future, similar to how it has become routine to refer all patients with Klebsiella bacteraemia to ophthalmologists for screening for endophthalmitis in our local hospitals.

## Background

*Elizabethkingia meningoseptica* (previously known as *Flavobacterium meningosepticum* and then *Chryseobacterium meningosepticum*) is a nosocomial non-fermenting gram-negative bacillus that has intrinsic resistance to many antibiotics commonly used in intensive care settings [1, 2]. In addition, it carries many antimicrobial resistance genes, as well as the ability to form biofilms and survive for long periods in a moist environment or in water sources including tap water, making it an important organism in many intensive care environments [3–5]. Unsurprisingly, outbreaks of this bacteria have been reported, including a recent publication reporting an outbreak in our local hospital’s cardiothoracic and surgical intensive care units (ICUs) in 2012 (Table 1) [6–10].

**Table 1:** **Summary of outbreaks of** ***Elizabethkingia meningoseptica***

Period of outbreak	Type of unit	Population involved	Source of outbreak	Control measures	Outcome
April to October 2002 [6]	Neonatal intensive care unit	4 neonates	Not found	Controlled by reinforcement of usual measures	No additional colonization/infection confirmed for >1 year after last case
July 2006 and January 2007 [7]	Neonatal intensive care unit and pediatric wards	8 newborns and 5 older children	Hand cultures obtained from a senior resident; Environmental cultures obtained from powdered infant formula, an electrical button, a computer keyboard, phone, a doorknob, and an Ambu bag	Staff exchange in wards restricted; All units thoroughly scrubbed using 2 disinfectants 3 times a day until outbreak controlled; Contact precautions.	Nine patients improved on antimicrobial treatment, and 4 premature infants died after infection.
December 2007 through April 2008 [8]	Long-term acute care hospital	19 patients with respiratory failure on mechanical ventilation	Environmental sampling: one swab out of 106 surfaces; Patient sampling: *E. meningoseptica* isolated from blood, respiratory specimen, catheter tip	Training on handwashing and disinfection practices, isolation policies, use of gowns and gloves, policies implemented regarding proper disposal of body fluids	Eight out of 19 died
Fall, 2006 [9]	Orthopaedic wards	2 patients who had allograft-associated surgical site infections	*E. meningoseptica* was recovered from sink drains and traps in clean rooms where tissues were processed	All clean-room sink drains and traps at processing facility replaced, check valves in drains installed, routine sanitization of drains started,	Tissue-processing resumed following these changes; sterility failure rates returned to baseline levels with no identification of E. meningoseptica or other waterborne gram-negative bacteria
August and September 2012 [10]	Intensive care units (ICUs).	5 patients	E. meningoseptica was isolated from from aerators, hand hygiene sinks	Urgent education programme instituted; Taps were cleaned systematically and aerators were changed.	Temporary reduction in case numbers achieved.

Endogenous bacterial endophthalmitis is a rare but serious condition that occurs when bacteria cross the blood–ocular barrier and multiply within the eye. While *E. meningoseptica* has long been recognized as a rare but serious cause of neonatal meningitis and sepsis, as well as an increasingly important pathogen in healthcare environments, its role as a cause of ocular pathology is not well-known [11]. So far, there have only been two reported cases of endophthalmitis related to *E. meningoseptica*, both of which were post-traumatic and hence, exogenous, in nature [12, 13]. We report the first case of *E. meningoseptica* endogenous endophthalmitis caused by bacteraemia by the same organism.

## Case presentation

A 75-year-old Chinese Singaporean lady with multiple comorbidities including Type 2 diabetes mellitus on insulin, hypertension, dyslipidemia, ischaemic heart disease, end-stage renal failure on hemodialysis, degenerative spondylosis and osteoarthritis, was admitted for fever, chills and rigors of one-day duration. There was also discharge of pus over her right femoral dialysis catheter.

She was diagnosed to have disseminated Methicillin-sensitive *Staphylococcus aureus* (MSSA) bacteraemia secondary to infected right femoral non-tunnelled dialysis catheter. Her first nine blood cultures grew MSSA, so did her wound culture. Her femoral line was removed and she was treated in medical intensive care unit (MICU) for her septic shock. She was referred to the infectious diseases (ID) team and she received systemic vancomycin, clindamycin and then cloxacillin over the course of several weeks as culture results became known. The patient subsequently improved clinically with resolution of fever and improvement of haemodynamic parameters. Her inflammatory markers including procalcitonin and white blood cell count showed decreasing trends. Approximately six weeks after admission, there was documented clearance of MSSA from her blood. The plan by ID was to continue intravenous cloxacillin for a total of six weeks from her last negative blood culture for the presumed endovascular infection although she had a normal two dimensional echocardiograph.

Unfortunately her stay in hospital was delayed as she developed extensive right lower limb deep vein thrombosis, for which she was treated with heparin infusion, inferior vena cava (IVC) filter and subsequently warfarin. Approximately two months into her stay in hospital, she developed an episode of sepsis with desaturation, drop in Glasgow coma scale (GCS) and low grade fever. Urgent neuroimaging revealed no intracranial infarct or hemorrhage. However her subsequent four blood cultures, taken two days apart, all grew *Elizabethkingia meningoseptica*, which was susceptible to cotrimoxazole, levofloxacin, and minocycline, but resistant to amikacin. She was treated with intravenous antibiotics, comprising courses of cotrimoxazole, cefazolin and meropenem, after consultation with the ID team. Three days after her first positive blood culture for *Elizabethkingia meningoseptica*, she developed a red and painful left eye with blurring of vision.

A referral to Ophthalmology was promptly made. On examination her right pupil was noted to be sluggish, and left pupil fixed. There was a reverse relative afferent pupillary defect (RAPD). Visual acuity in her right eye was 6/120 (without reading glasses) with no perception of light (NPL) in her left eye. Examination of her left eye showed an injected conjunctiva, hazy cornea with Descemet’s membrane (DM) folds, and a deep anterior chamber filled with a hypopyon and fibrin. A glint of an intraocular lens could be seen. There was no view of the fundus but B-scan ultrasonography showed multiple vitreous opacities. The impression was that of left eye endogenous endophthalmitis, for which she underwent an intravitreal tap for vitreous culture, as well as injection of intravitreal vancomycin and amikacin. She was also started on hourly fortified gentamicin and cefazolin eyedrops. In view of the absence of perception of light, as well as her systemic status, vitrectomy was not resorted to. There was also no immediate indication for evisceration.

The patient’s vitreous cultures came back positive for *Elizabethkingia meningoseptica*, which was sensitive to ciprofloxacin, levofloxacin, cotrimoxazole, and minocycline, but resistant to ceftazidine, gentamicin and amikacin. In consideration of the sensitivity results, the intravitreal antibiotics were switched to ciprofloxacin (100 micrograms/0.05 ml). The injection was repeated five times before it was felt that the infection was under reasonable control. The fibrin clot in her anterior chamber progressively resolved and the inflammatory material in the vitreous cavity became organised. The eye was quiet with no pain. While vision was not expected to recover, the need for evisceration was successfully averted.

Potential environmental source links were investigated. Workflows for ward staff were reviewed. Environmental screening was performed by swab cultures of surfaces and equipment within patient rooms, taps and aerators, water sampling and sampling of chlorhexidine solution. While no clear source of the bacteria was identified, it was presumably from aerators and hand hygiene sinks, as was found in a previous investigation of the same bacteria in the hospital [10].

## Discussion

*Elizabethkingia menigoseptica* has reduced susceptibility to a broad range of antimicrobials, including beta lactams, aminoglycosides and chloramphenicol. It is an opportunistic pathogen being most often associated with meningitis and septicemia in a pediatric (especially neonatal) population. It has also been identified as a cause of healthcare-associated pneumonia, sepsis and meningitis in adults, particularly in immunocompromised patients, those with indwelling central venous catheters and those with long hospital stays. Mortality in patients with *E. meningoseptica* bacteraemia or meningitis may be high (>50%) depending on the population studied [7, 14, 15].

To date, there have only been two case reports on *E. meningoseptica*-related endophthalmitis, both exogenous in nature, in contrast to our patient who had an endogenous cause [12, 13]. One was a case of deliberate ocular penetration with an unsterile sewing needle in a patient with depressive illness, while the other was a case of penetrating eye injury caused by a truck tyre explosion resulting in a self-sealing full thickness corneal wound with two metallic posterior segment foreign bodies [13]. While the above two cases differ from ours in the nature of the infection, there are similarities in all cases: the infection in the eye occurred acutely, progressed very quickly from one to three days, behaved virulently and produced an intense fibrinous reaction in the anterior and posterior segments of the eye. *E. meningoseptica* cultured from the vitreous, like those from other parts of the body, was found to be resistant to many antibiotics. Ciprofloxacin was one antibiotic to which it was sensitive to in all three cases, and hence may be considered as first-line therapy (systemic and intravitreal) in future cases of *E. meningoseptica* related endophthalmitis.

There have been no previous reports of *E. meningoseptica* causing endogenous endophthalmitis, which occurs when organisms reach the eye via the bloodstream, and enter the internal ocular spaces by crossing the blood–ocular barrier. Endogenous bacterial endophthalmitis is treated seriously because they are serious infections with poor visual outcomes [16]. The outcomes worsen with several factors: delay in diagnosis, use of inappropriate antibiotics, diffuse infection of the vitreous and retina or panophthalmitis, poor vision at presentation, infection with virulent organisms and gram negative infection [16]. Gram negative endophthalmitis is more common in East Asian hospitals, with the most common bacteria being Klebsiella spp, *Escherichia coli, Pseudomonas aeruginosa*, *Neisseria meningitides* and *Serratia marscescens*. In fact because of its virulence and prevalence, it has become routine, in our local hospitals, to refer all patients with Klebsiella bacteraemia to ophthalmologists for screening for endophthalmitis.

*Elizabethkingia menigoseptica*, like Klebsiella spp, has shown to behave in a virulent manner in the eye. With its increasing prevalence in health care settings, especially in intensive care environments due to its propensity to form biofilms, it is of utmost importance for physicians to have a high vigilance for this opportunistic pathogen. While its presence in the blood may not necessarily require an automatic referral for eye screening, they should consider an early eye referral if the patient has any signs (conjunctival injection or chemosis, periocular redness and swelling, hazy cornea, hypopyon in anterior chamber, reduced or absent red reflex) or symptoms (floaters, blurring of vision, eye pain, tearing, redness) of an eye infection. However, in view of the aggressiveness and virulence of this bacteria, and the likelihood of misdiagnosis or missed diagnosis of endogenous endophthalmitis especially given its low incidence, we may want to consider screening all cases of *E. menigoseptica* bloodstream infections for endophthalmitis in future.

This case also brings up the issue of potential contamination of taps and aerators within ICUs as important sources of *E. meningoseptica* infection as may have been in this case [6, 8, 10]. *E. meningoseptica* is an opportunistic pathogen that is well adapted to the intensive care environment due to its intrinsic resistance to antimicrobials commonly used in the ICU setting and its propensity to form biofilms. The latter are difficult to eradicate, particularly where the organism has established on an indwelling device such as a vascular line or endotracheal tube.

Previous outbreaks have shown a multitude of potential sources of the bacteria, including sink drains, taps, aerators, catheters, blood and respiratory specimens, hand cultures, infant formula, electrical buttons, a computer keyboards, phone, doorknobs, Ambu bags etc. [6–10]. Beyond the potential contribution of *E. meningoseptica* to the deterioration of the severely ill adult patient, transmission of *E. meningoseptica* from environmental reservoirs to neonates is a known infection outbreak scenario. Infection control personnel in acute care hospitals must be aware of *E. meningoseptica* in chronically ill patients, especially those on mechanical ventilation in ICUs. Attention should also be paid to non-adherence to existing policies regarding disposal of patient secretions and cleaning of re-usable patient care items, time pressures and distance between patient rooms and ward utility rooms notwithstanding.

Design of wards particularly within the critical care setting must consider physical and personnel requirements to facilitate safe delivery of care, and staff workflows must be optimized to facilitate patient safety and avoid inadvertently putting patients at risk with lapses in safety procedures [10].

## Conclusion

In conclusion, the incidence of endogenous endophthalmitis by *E. meningoseptica* and other bacteria is reportedly low, but clinicians must not fail to appreciate the overlap between ocular and systemic disease. Prompt diagnosis and treatment is essential if useful vision is to be preserved and therefore ophthalmologists, physicians, and microbiologists need to have a high level of awareness of this disease to reduce harm to our patients.

## Consent

Written informed consent was obtained from the patient for publication of this Case report and any accompanying images. A copy of the written consent is available for review by the Editor-in-Chief of this journal.
